# Circumpolar dataset of sequenced specimens of *Promachocrinus kerguelensis* (Echinodermata, Crinoidea)

**DOI:** 10.3897/zookeys.315.5673

**Published:** 2013-07-04

**Authors:** Lenaïg G. Hemery, Nadia Améziane, Marc Eléaume

**Affiliations:** 1 Muséum national d’Histoire naturelle, Département des Milieux et Peuplements Aquatiques, UMR 7208-MNHN, UPMC, CNRS, IRD-207, CP26, 57 rue Cuvier, 75231 Paris Cedex 05, Paris, France

**Keywords:** Antarctica, Crinoidea, Cytochrome Oxydase subunit I, Echinodermata, Phylogeography, *Promachocrinus*, Southern Ocean, Sub-Antarctic

## Abstract

This circumpolar dataset of the comatulid (Echinodermata: Crinoidea) *Promachocrinus kerguelensis* (Carpenter, 1888) from the Southern Ocean, documents biodiversity associated with the specimens sequenced in Hemery et al. (2012). The aim of Hemery et al. (2012) paper was to use phylogeographic and phylogenetic tools to assess the genetic diversity, demographic history and evolutionary relationships of this very common and abundant comatulid, in the context of the glacial history of the Antarctic and Sub-Antarctic shelves (Thatje et al. 2005, 2008). Over one thousand three hundred specimens (1307) used in this study were collected during seventeen cruises from 1996 to 2010, in eight regions of the Southern Ocean: Kerguelen Plateau, Davis Sea, Dumont d’Urville Sea, Ross Sea, Amundsen Sea, West Antarctic Peninsula, East Weddell Sea and Scotia Arc including the tip of the Antarctic Peninsula and the Bransfield Strait. We give here the metadata of this dataset, which lists sampling sources (cruise ID, ship name, sampling date, sampling gear), sampling sites (station, geographic coordinates, depth) and genetic data (phylogroup, haplotype, sequence ID) for each of the 1307 specimens. The identification of the specimens was controlled by an expert taxonomist specialist of crinoids (Marc Eléaume, Muséum national d’Histoire naturelle, Paris) and all the COI sequences were matched against those available on the Barcode of Life Data System (BOLD: http://www.boldsystems.org/index.php/IDS_OpenIdEngine). This dataset can be used by studies dealing with, among other interests, Antarctic and/or crinoid diversity (species richness, distribution patterns), biogeography or habitat / ecological niche modeling. This dataset is accessible through the GBIF network at http://ipt.biodiversity.aq/resource.do?r=proke.

## Project details

**Project title:** Comprehensive sampling reveals circumpolarity and sympatry in seven mitochondrial lineages of the Southern Ocean crinoid species *Promachocrinus kerguelensis* (Echinodermata)

**Personnel:** Lenaïg G. Hemery

**Funding:** French ANR ANTFLOCKS (n° 07-BLAN-0213-01); MNHN Paris intern grants (DMPA’s BQR, ATMs “Biominéralisation”; “Biodiversité actuelle et fossile; crises, stress, restaurations et panchronisme: le message systématique”; “Taxonomie moléculaire: DNA Barcode et gestion durable des collections”).

**Study area descriptions/descriptor:** The 1307 specimens in this dataset were collected from the Southern Ocean, south of the Sub-Antarctic Front (SAF): Kerguelen Plateau (Kerguelen and Heard islands), Davis Sea, Dumont d’Urville Sea, Ross Sea, Amundsen Sea, West Antarctic Peninsula, East Weddell Sea and Scotia Arc (from the tip of the Antarctic Peninsula and the Bransfield Strait to the South Georgia island). The bathymetric range extended from 65 to 1162 meters deep.

**Figure 1.**
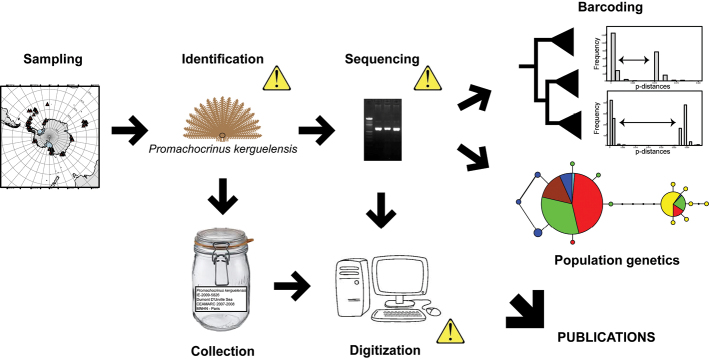
Synoptic of the procedure used to generate the dataset. Yellow exclamatory marks show where the data quality has been checked.

**Design description:** This dataset was gathered to conduct a circumpolar phylogeographic study of the crinoid species *Promachocrinus kerguelensis* (Hemery et al. 2012) and designed to spatially improve the sampling of Wilson et al. (2007), which was limited to the Atlantic sector of the Southern Ocean. The aim of Hemery et al. (2012) was to test the circumpolarity of the genetic lineages of Wilson et al. (2007), and to test whether these lineages represented an under-sampling artifact of a large and genetically diverse metapopulation or whether they were truly representative of the Southern Ocean. The authors used a sampling strategy designed to cover the broadest possible genetic variation and to explore the evolutionary relationships among the seven lineages, in order to be able to conduct population analyses (Meyer and Paulay 2005). They also wanted to understand the distributional limits of each phylogroup in *Promachocrinus kerguelensis* to assess the connectivity displayed throughout their range, and to test the “multiple refugia” theory by studying the demographic history of each phylogroup. For this purpose, more than two thousand specimens, sampled during the most recent Antarctic cruises focused on benthic biodiversity and fixed and preserved in a way allowing for DNA extraction and amplification (fixed in ethanol or frozen), were provided by several taxonomists and benthologists from different institutions. Specimen identifications during the sampling cruises were conducted to a higher level allowed by the taxonomic skills of the collectors then checked principally at the Muséum national d’Histoire naturelle, Paris by taxonomists trained to deal with Antarctic crinoids. The Cytochrome c Oxydase subunit I (COI) was successfully sequenced for 1307 of these specimens. Both collection data and produced sequences were digitized in appropriate databases, used or ready to be used for publishing purpose (Figure 1).

**Data published through GBIF:** http://ipt.biodiversity.aq/resource.do?r=proke as an Excel spreadsheet of the dataset, available through the Darwin Core Archive format at http://ipt.biodiversity.aq/archive.do?r=proke.

## Taxonomic coverage

**General taxonomic coverage description:** This dataset focuses on the Antarctic comatulid species *Promachocrinus kerguelensis* (Carpenter 1888), the most abundant and morphologically variable comatulid species in the Southern Ocean (Speel and Dearborn 1983). It corresponds to the 1307 specimens sequenced in Hemery et al. (2012).

## Taxonomic ranks

**Phylum:** Echinodermata

**Class:** Crinoidea

**Order:** Comatulida

**Family:** Antedonidae

**Subfamily:** Heliometrinae

**Genus:** *Promachocrinus*

**Species:** *kerguelensis*

**Common names**: echinoderm, crinoid, comatulid, feather star

## Spatial coverage

### General spatial coverage

**Figure 2.**
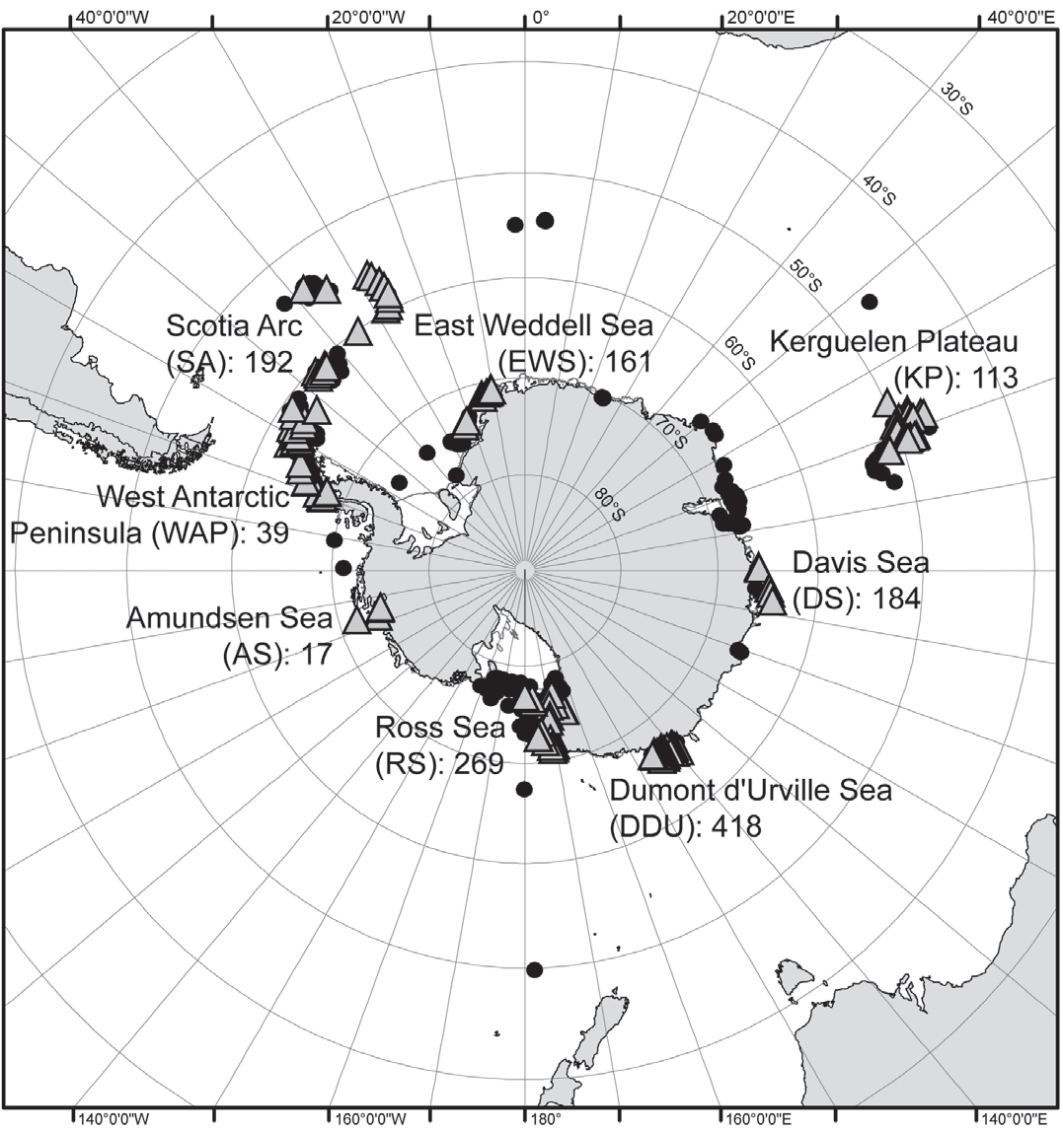
*Promachocrinus kerguelensis* sampling stations in the Southern Ocean. Triangles represent the sampled stations, circles represent the bibliographic data, numbers are sequenced specimens per region (modified from Hemery et al. 2012).

The specimens of *Promachocrinus kerguelensis* gathered in this dataset were collected from most of the strategic regions in the Southern Ocean (triangles in Figure 2): the Antarctic continental shelf (East Weddell Sea, Davis Sea, Dumont d’Urville Sea, Ross Sea, Amundsen Sea, West Antarctic Peninsula), the Scotia Arc islands (South Shetland, South Orkney and South Sandwich) and the Sub-Antarctic islands (South Georgia, Kerguelen and Heard). Specimens were sampled at depths ranging from 65 m to 1162 m. This covers most of the known distribution area of this species (black circles in Figure 2), but only a portion of the bathymetric range for this species, which extends from 10 m to 2100 m (Speel and Deardorn 1983).

### Coordinates

76°49'58.8"S and 47°12'14.4"S Latitude; 107°24'28.8"W and 170°23'6"E Longitude.

### General temporal coverage

**Figure 3.**
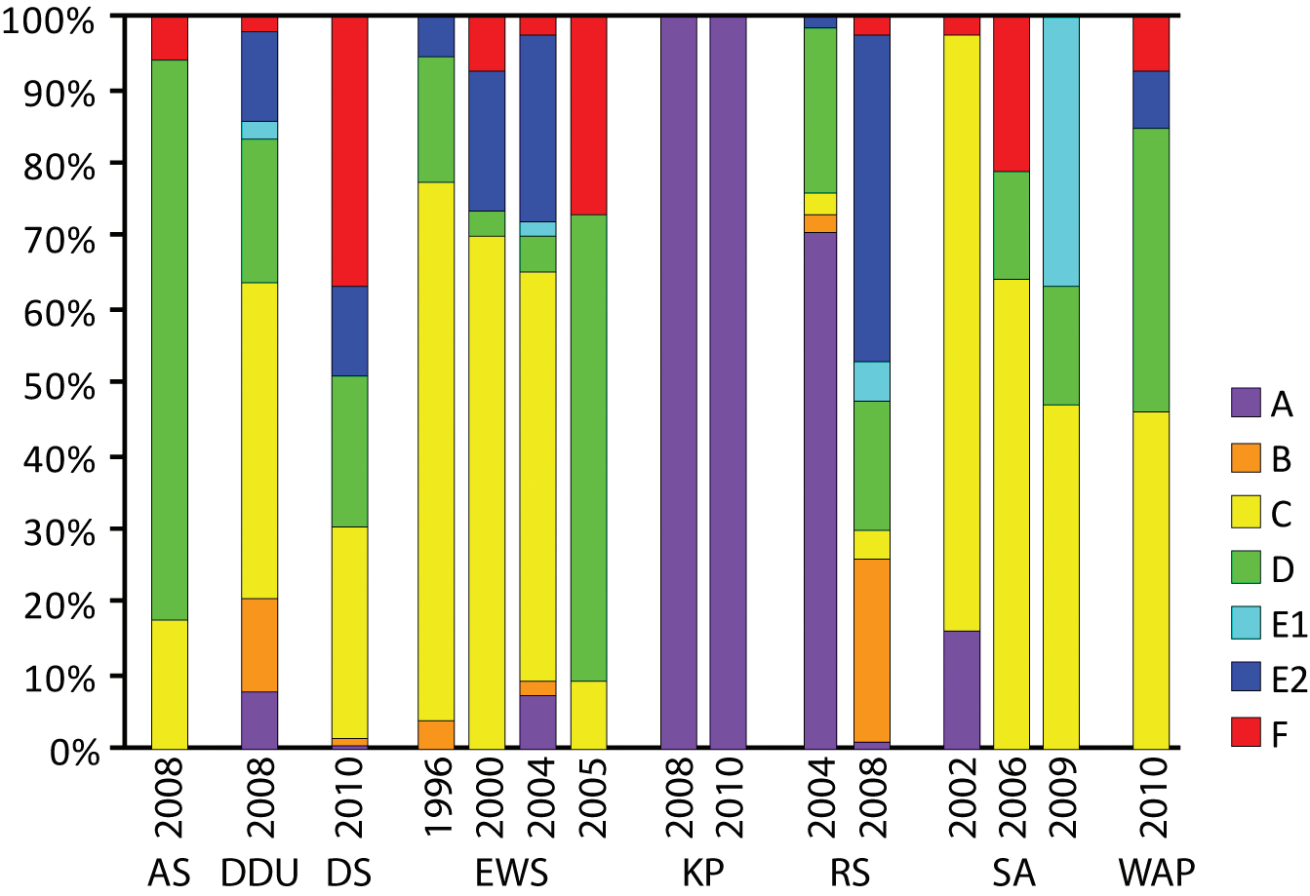
Proportion of specimens per phylogroup per year and sampling region. Acronyms of regions are given in Figure 2, A - F are the names of the 7 COI haplogroups found in Hemery et al. 2012.

The specimens were collected during one to four different cruises per sampling region for a total of 17 cruises from 1996 to 2010 (Figure 3). However, the number of specimens was too variable among cruises to be statistically compared (see details of numbers in the Methods part).

**Temporal coverage:** January 26, 1996 – March 16, 1996

**Temporal coverage:** March 18, 2000 – May 11, 2000

**Temporal coverage:** January 23, 2002 – May 5, 2002

**Temporal coverage:** November 17, 2003 – January 19, 2004

**Temporal coverage:** January 15, 2004 – March 15, 2004

**Temporal coverage:** February 9, 2004 – February 22, 2004

**Temporal coverage:** January 20, 2005 – April 7, 2005

**Temporal coverage:** February 27, 2006 – April 11, 2006

**Temporal coverage:** December 16, 2007 – January 27, 2008

**Temporal coverage:** February 18, 2008 – April 11, 2008

**Temporal coverage:** January 31, 2008 – March 16, 2008

**Temporal coverage:** February 6, 2009 – March 12, 2009

**Temporal coverage:** December 1, 2009 – December 11, 2009

**Temporal coverage:** December 29, 2009 – January 8, 2010

**Temporal coverage:** August 28, 2010 – September 28, 2010

## Natural collections description

**Parent collection identifier:** Muséum national d’Histoire naturelle, Paris (MNHN)

**Collection name:** Echinodermes (MNHN-IE)

**Collection identifier:** Marc Eléaume

**Specimen preservation method:** Alcohol

## Methods

**Method step description:** See sampling description below and graphic summary in Figure 1.

**Study extent description:** The specimens were collected during 17 cruises in the Southern Ocean, from 1996 to 2010 (Figures 2 and 3): 53 specimens from EASIZ I (ANT XIII/3) onboard the RV *Polarstern* (1996) in the East Weddell Sea, 53 from EASIZ III (ANT XVII/3) onboard the RV *Polarstern* (2000) in the East Weddell Sea and the Scotia Arc, 12 from ANDEEP I&II (ANT XIX/3&4) and 25 from LAMPOS (ANT XIX/5) onboard the RV *Polarstern* (2002) in the Scotia Arc, 43 from BENDEX (ANT XXI/2) onboard the RV *Polarstern* (2003-2004) in the East Weddell Sea, 15 from ITALICA 2004 onboard the RV *Italica* (2004) in the Ross Sea, 106 from TAN0402 onboard the RV *Tangaroa* (2004) in the Ross Sea, 12 from ANDEEP III (ANT XXII/3) onboard the RV *Polarstern* (2005) in the East Weddell Sea, 14 from BIOPEARL I (JR144) onboard the RV *James Clark Ross* (2006) in the Scotia Arc, 418 from CEAMARC (2007/08 V3) onboard the RV *Aurora Australis* (2007-2008) in the Dumont d’Urville Sea, 17 from BIOPEARL II (JR179) onboard the RV *James Clark Ross* (2008) in the Amundsen Sea, 2 from HIMI-SC50 onboard the FV *Southern Champion* (2008) on the Kerguelen Plateau (Heard island), 148 from TAN0802 onboard the RV *Tangaroa* (2008) in the Ross Sea, 68 from AMLR 2009 Leg II onboard the RV *Yuzhmorgeologiya* (2009) in the Scotia Arc and the West Antarctic Peninsula, 26 from BASWAP (JR230) onboard the RV *James Clark Ross* (2009) in the West Antarctic Peninsula, 184 from BR09 onboard the RV *Aurora Australis* (2009-2010) in the Davis Sea, and 111 from POKER II onboard the FV *Austral* (2010) on the Kerguelen Plateau (Kerguelen island).

**Sampling description:** The specimens were sampled using several sampling gears, depending on the cruise: agassiz trawls, beam trawls, bottom trawls, box corers, epibenthic sledges (Arntz and Brey 2001, 2003, 2005; Arntz and Gutt 1997; Beaman and O’Brien 2009; Duhamel et al. 2011; Fahrbach 2006; Fütterer et al. 2003; Lockhart et al. 2009). During each cruise, specimens were sorted onboard and then fixed and preserved in 70–95% ethanol or first frozen and subsequently preserved in ethanol. The specimens were curated by each institution once back from the field and digitized in their own databases before the specimens were gathered by the authors in the purpose of the molecular study. Metadata associated with each specimen were extracted from the cruise reports. The molecular data (barcoding) were generated following the protocols described in Ivanova et al. (2006), Eléaume et al. (2011) and Hemery et al. (2012).

**Quality control description:** The initial geo-referencing was done by means of the vessel onboard GPS systems. Samples identification was supervised and checked by Marc Eléaume, crinoid taxonomist at the Muséum national d’Histoire naturelle, Paris, following Clark and Clark (1967) taxonomic description of the species, and matched to the World Register of Marine Species (WoRMS). The barcoding was done by Lenaïg G. Hemery at the Muséum national d’Histoire naturelle, Paris, and by the Canadian Center for DNA Barcoding, Toronto, and the Scripps Institution of Oceanography, San Diego, and matched to sequences already available on the Barcode of Life Data System (BOLD: http://www.boldsystems.org/index.php/IDS_OpenIdEngine). All sequences, specimen occurrences and identifications are linked together through unique numbers in BOLD under the public project name PROKE.

## Datasets

**Dataset description:** This dataset has been generated for a molecular study of the Antarctic comatulid species *Promachocrinus kerguelensis*, improving the geographic coverage of the previous study by Wilson et al. (2007). All the specimens are identified by several types of numbers that are linked together: Sample ID (characteristic of each individual), BOLD ID, GenBank ID and SeqID (all three characteristic of each sequence in different databases), Field Number (when available) and Museum ID. In some cases, the two last identifiers are shared by several individuals identifiable from each other by their own Sample ID. The dataset also includes the name of the institution storing the specimens, the complete taxonomy, names of identifiers and collectors, and information on the sampling itself: cruise names, vessel names, sampling gears, dates, regions, sectors, exact sites (when available), station numbers, latitudes and longitudes in decimal degrees, and depths in meters. This dataset is suitable to be used in studies dealing with, for example, Antarctic and/or crinoid diversity (species richness, distribution patterns), biogeography or habitat / ecological niche modeling.

**Object name:** Darwin Core Archive Circumpolar dataset of sequenced specimens of *Promachocrinus kerguelensis* (Echinodermata, Crinoidea)

**Character encoding:** UTF-8

**Format name:** Darwin Core Archive format

**Format version:** 1.0

**Distribution:** http://ipt.biodiversity.aq/archive.do?r=proke

**Publication date of data:** 2012-03-01

**Language:** English

**Metadata language:** English

**Date of metadata creation:** 2012-04-25

**Hierarchy level:** Dataset

## Appendix

Sampling data associated to the 1307 specimens of *Promachocrinus kerguelensis* sequenced in Hemery et al. (2012). (doi: 10.3897/zookeys.315.5673.app) File format: Comma-Separated Values File (csv). **Explanation note:** Each sample is associated to three unique sequence IDs (BOLD ID, GenBank ID and SeqID); Haplotype and Clade refer back to the genetic data from Hemery et al. (2012); Latitude and Longitude are given in decimal degrees; Depth is given in meters.
